# Supplementary material for: Quantum scale organic semiconductors for SERS detection of DNA methylation and gene expression
Source: Nat Commun. 2020 Feb 28;11:1135. doi: 10.1038/s41467-020-14774-3 (PMC7048788; doi:10.1038/s41467-020-14774-3)
Supplement: Supplementary file 1 — Supplementary Information [file 41467_2020_14774_MOESM1_ESM.docx]

**Quantum scale organic semiconductors for SERS detection of DNA methylation and gene expression**

**Authors**

*Swarna Ganesh ^b, c, e^, Krishnan Venkatakrishnan, ^a,c,e *^ Bo Tan ^a^,^d ,e^*

^a^ Affiliate Scientist, Keenan Research Center, St. Michael’s Hospital, 209 Victoria Street, Toronto, Ontario, M5B 1T8, Canada

^b^ Institute for Biomedical Engineering, Science and Technology (I BEST), Partnership between Ryerson University and St. Michael’s Hospital, Toronto, Ontario M5B 1W8, Canada

^c^ Ultrashort Laser Nanomanufacturing Research Facility, Department of Mechanical and Industrial Engineering, Ryerson University, 350 Victoria Street, Toronto, ON M5B 2K3, Canada

^d^ Nanocharacterization Laboratory, Department of Aerospace Engineering, Ryerson University, 350 Victoria Street, Toronto, Ontario M5B 2K3, Canada.

^e^ Nano Bio Interface facility, Department of Mechanical and Industrial Engineering, Ryerson University, 350 Victoria Street, Toronto, ON M5B 2K3, Canada.

**Corresponding Author:** venkat@ryerson.ca


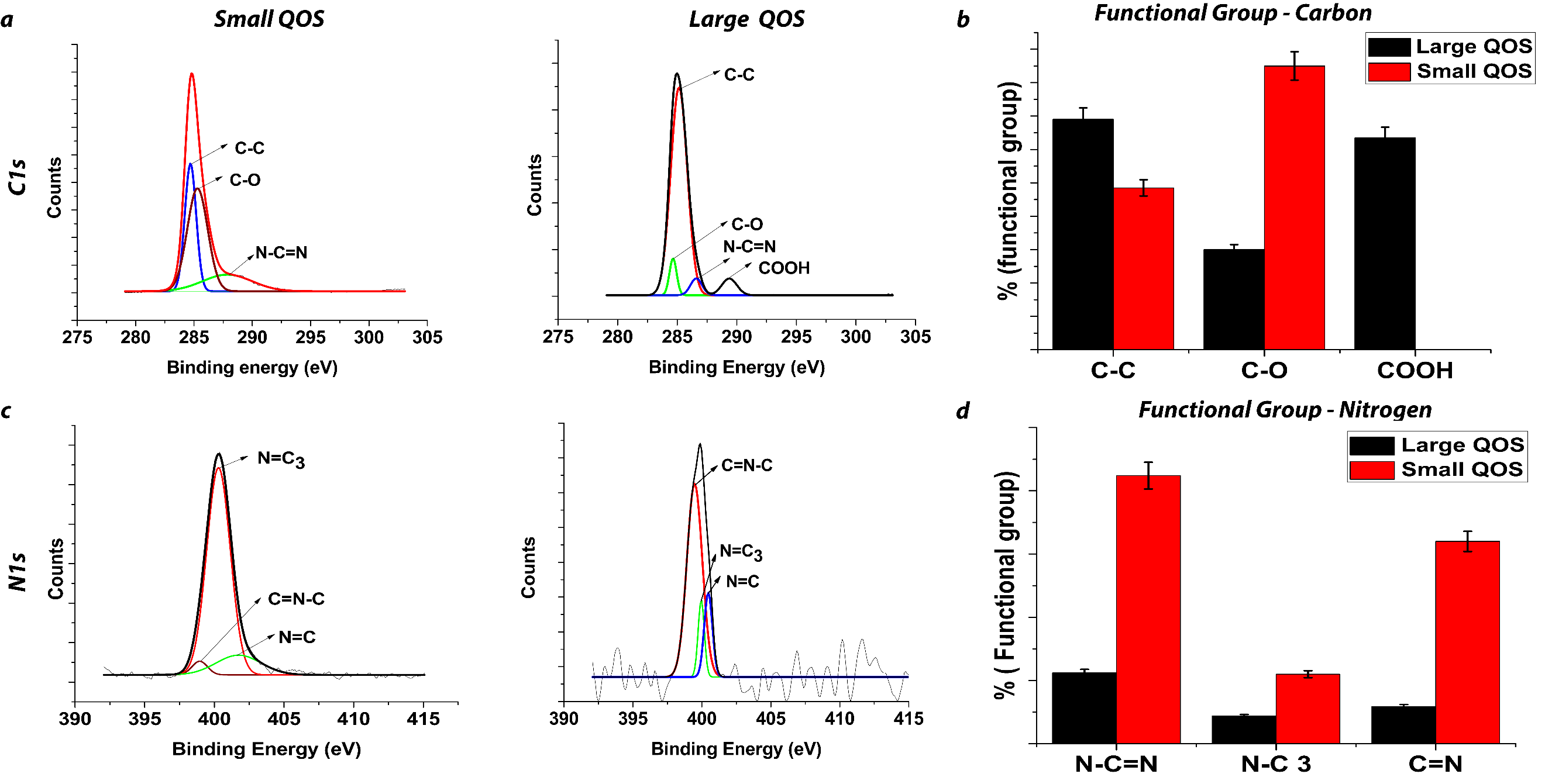


**Supplementary Figure 1: Structural and chemical characterizations of QOS a) C1s XPS spectra determining the density of carbon functional groups in QOS, b) Comparison of carbon functional groups between Small QOS and Large QOS, c) N1s XPS spectra determining the density of Nitrogen functional groups in QOS, d) Comparison of carbon functional groups between Small QOS and Large QOS**

**Supplementary Note 1**: XPS analysis revealed the chemical and structural composition of QOS. The C1s spectra revealed the4 peaks centering 284.6 eV, 286.1eV, 288 eV and 289.2 eV corresponding to sp2 C-C bonds, C-O bond, N-C=N and COOH bond, respectively. The N1s spectra exhibited three peaks at 398.8 eV,399.8 eV and 400.4 eV associated with C=N-C, C-N-C and N-C_3_ , respectively ^1^. The carbon bonding arrangement suggested that most of the oxygen functionalities were present on the edge sites of QOS.


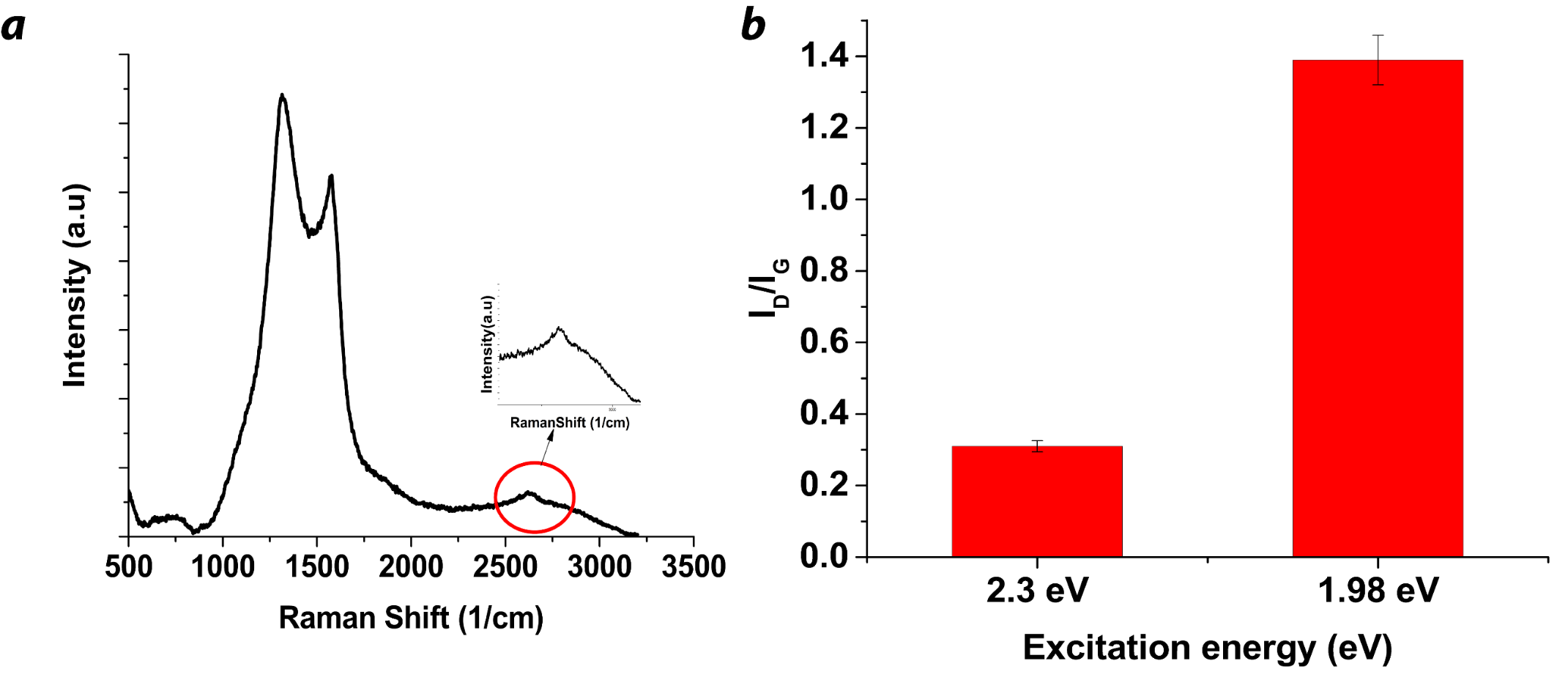


**Supplementary Figure 2 Raman spectroscopic characterization of QOS a) Raman spectra to determine the optical properties of QOS b) I_D_/I_G_ ratio as a function of laser excitation energy to determine the crystallite size of QOS**

**Supplementary Note 2:** The Raman peak at 1580 cm-1 corresponds to doubly degenerate E_2g_ phonons at the brillouin zone due to first-order Raman scattering process^2^. The substitution of nitrogen atom in graphene backbone results in the activation of defect induced double resonance process peak at 1318 cm-1^3^. The I_d_/I_g_ ratio is calculated to be 1.39 for laser excitation energy of 1.98eV and 0.31 for laser excitation energy of 2.3 eV. The crystallite size was calculated to be 65.5 nm and 62.01 nm. The crystallite size corresponds to a doping level of 0.26. The crystallite size decreases with increasing nitrogen defects in the particle. The intensity of 2D band of QOS is lower compared to D band and G band could be attributed to enhanced scattering effect from the nitrogen induced electron transport^4^. The I_2D_/I_G_ ratio depends on the electron concentration. In addition, the charge concentration significantly affects the interaction between optical phonons and the Dirac fermions^4^.


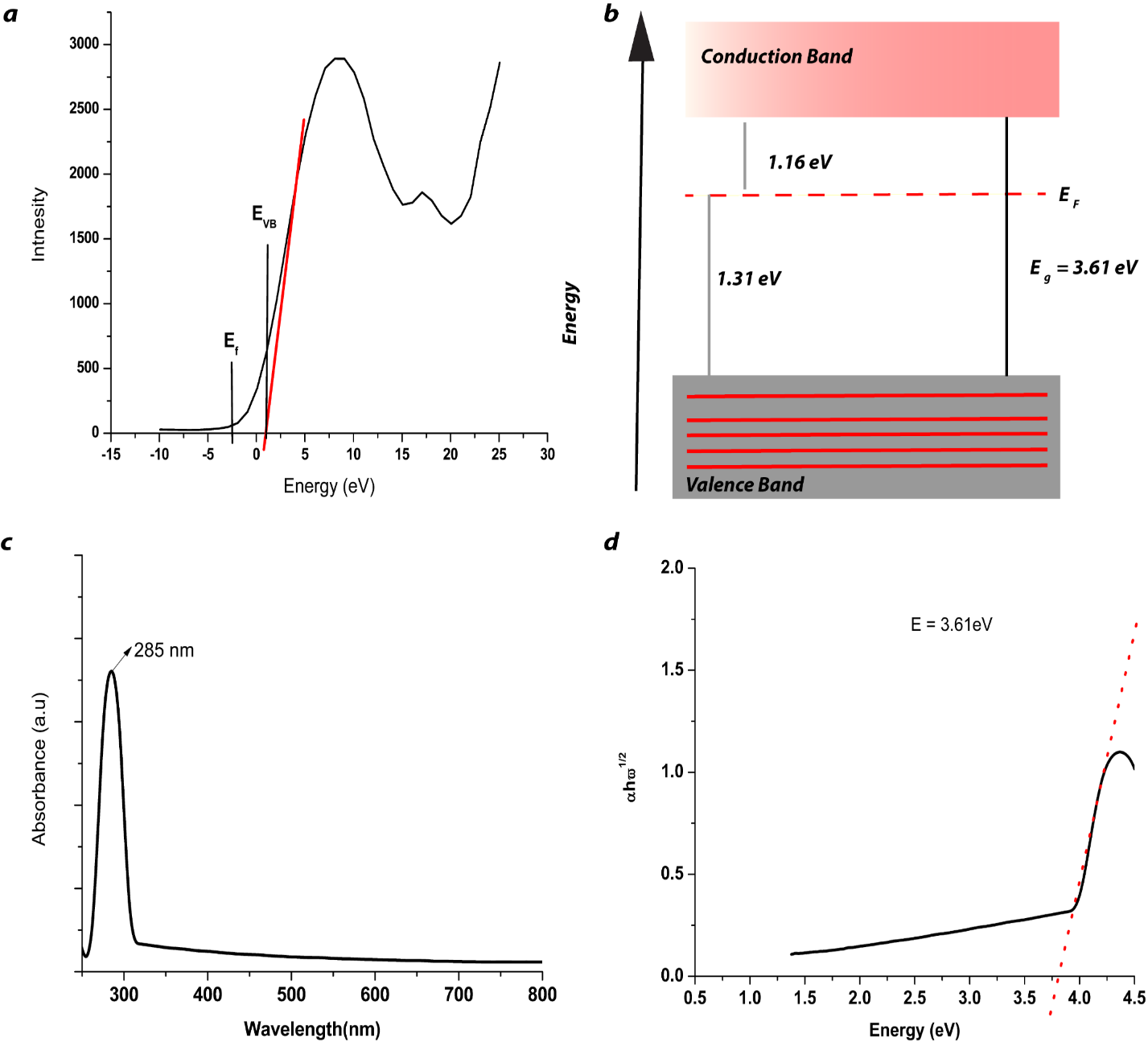


**Supplementary Figure 3: Bandgap Engineering in QOS a) Valence band spectrum of QOS to resolve the band structure b) Proposed band structure of QOS c) UV-visible spectra of QOS showcasing the absorption maximum in the UV range d) Tauc plot showing an indirect bandgap of 3.61eV**

**Supplementary Note 3:** The UV-Visible spectrum, given in Figure S3 c, shows an absorption maximum at 285nm ascribed to π-π^*^ transition of C=C. The optical absorption edge was observed to be at 365nm, extending into the visible region. This confirms the presence of n- π^*^ transition. This transition confirms the presence of heteroatoms in the graphene lattice which acts like electron donor. The change in atomic composition further leads to the increase of fermi energy level and the decrease in the effective π ^*^ orbital level ^5^. The optical band gap of organic semiconductor can be predicted from a Tauc plot. The Tauc plot is the relationship between (αhυ) ^r^ versus hυ obtained from the UV-Vis spectrum, where α, υ, h are the absorption coefficient, light frequency and Planck’s constant, respectively. The Tauc plot shows a linear fit for r = ½, suggesting the quantum organic semiconductor probe is an indirect bandgap material ^6^. The E_g_ value of QOS was thus determined to be 3.61eV by quantifying the X-axis intercept, shown in Figure S3d. When nitrogen atom is introduced in the graphene backbone, the density of state near the fermi level is inhibited, resulting in opening of band gap between the valence and conduction band.

The valence band spectrum in figure 3a was obtained through XPS to determine the band structure of the organic semiconductor probes. The valence band energy (E – VB) appears at 1.31 eV from the fermi level. The energy gap between the fermi energy level (E_f_) and conduction band is 1.16 eV. The significantly small gap between the conduction band and fermi level explains the extension of Tauc plot (figure S3d) into the visible region ^6^. The presence of multiple prominent peaks in the VB spectrum indicates the influence of numerous electron transitions. The most prominent peak 8.2eV is assigned to 2p σ- electrons that forms the carbon - carbon lattice ^5^. The broadening of C-C peak can be attributed to the presence of σ bonding oxygen atoms resulting in C-O bonds. Altering the onset of valence band can be accomplished by introducing non-bonding electrons in the C-C backbone ^7^. The peak at 17.06 eV is characteristic of C-N bonding as reported in literature ^6^


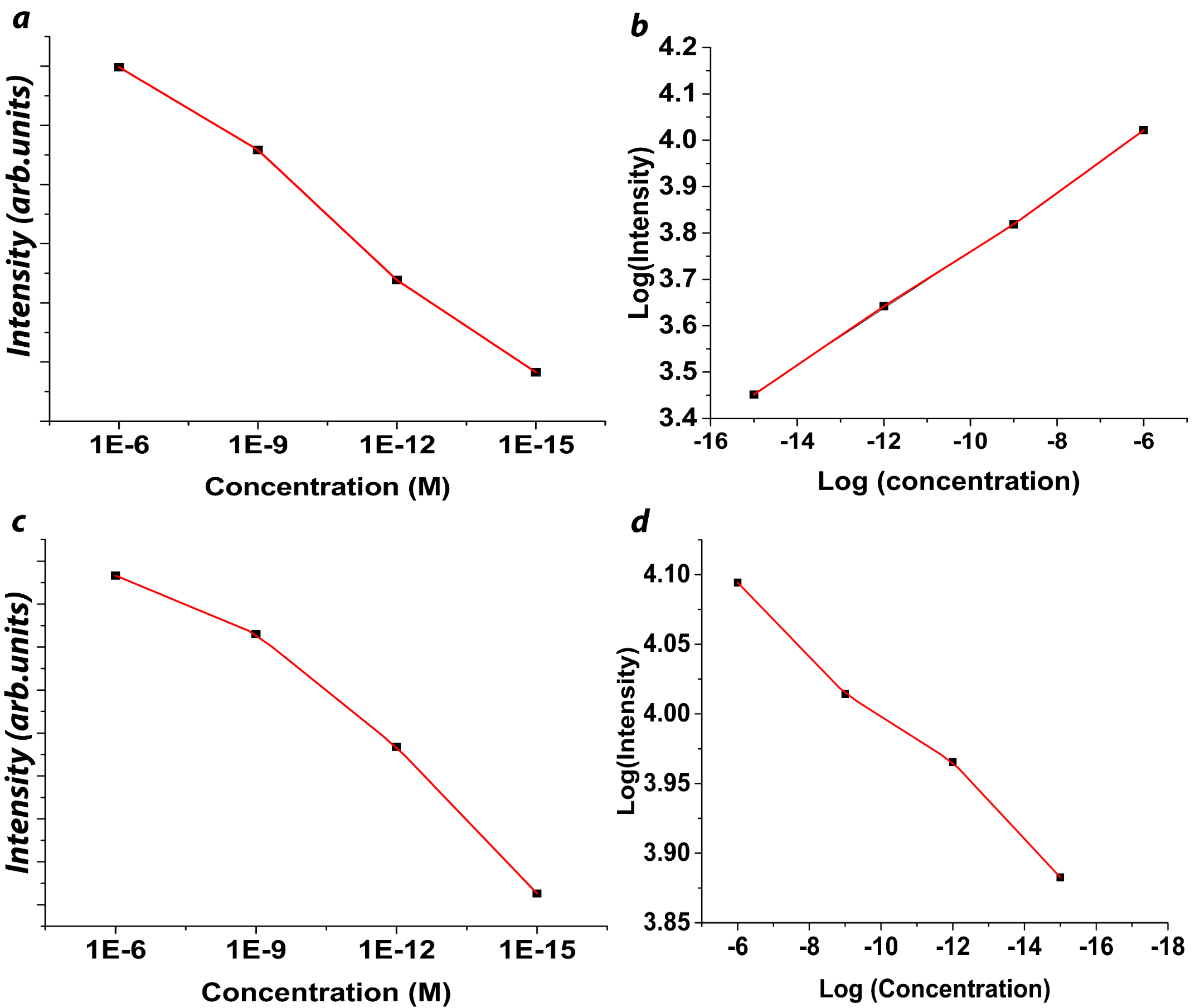


**Supplementary Figure 4: a), b) Limit of Detection for Crystal violet on QOS at various CV concentration as a function of intensity and log(intensity) respectively. c) d) Limit of detection for R6G on QOS at various R6G concentration as a function of intensity and log(intensity) respectively**

**Supplementary Table 1: SERS enhancement factors of CV and R6G at different concentrations**

| Molar Concentration | CV | R6G |
| --- | --- | --- |
| 1.00E-06 | 3.91E+10 | 4.85E+10 |
| 1.00E-09 | 3.23E+10 | 4.56E+10 |
| 1.00E-12 | 1.11E+12 | 4.57E+12 |
| 1.00E-15 | 1.39E+12 | 4.43E+12 |


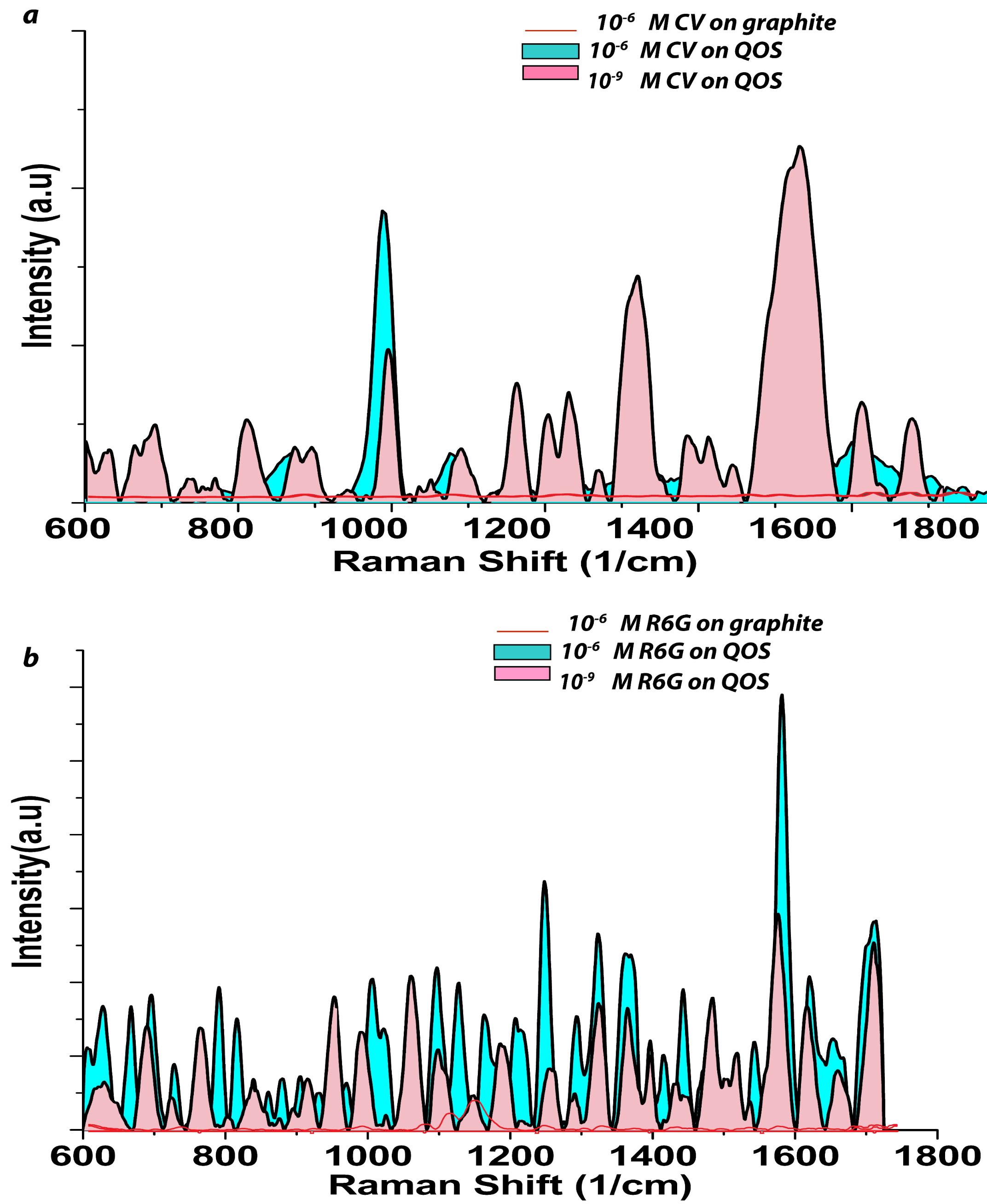


**Supplementary Figure 5:** **a) SERS spectra of 10^-6^ M and 10 ^-9^ M concentration of CV on QOS b) SERS spectra of 10^-6^ M and 10 ^-9^ M concentration of R6G on QOS**

**Supplementary Note 4 :** The mechanism of SERS enhancement is always a combined effect of plasmon resonance and charge transfer. The charge transfer process in the molecule-probe system involves various quantum states, including charge transition in the molecular level and exciton resonances. Even though the excitation wavelength is resonant with one of these processes, a considerable amount of enhancement may be attributed to intensity borrowing from the near field resonance processes^8^ . By utilizing the Herzberg-Teller correction to Born-Oppenheimer approximation, it could be concluded that SERS enhancement in a semiconductor system is a product of three resonances, namely: surface plasmon, charge-transfer and molecular resonance.


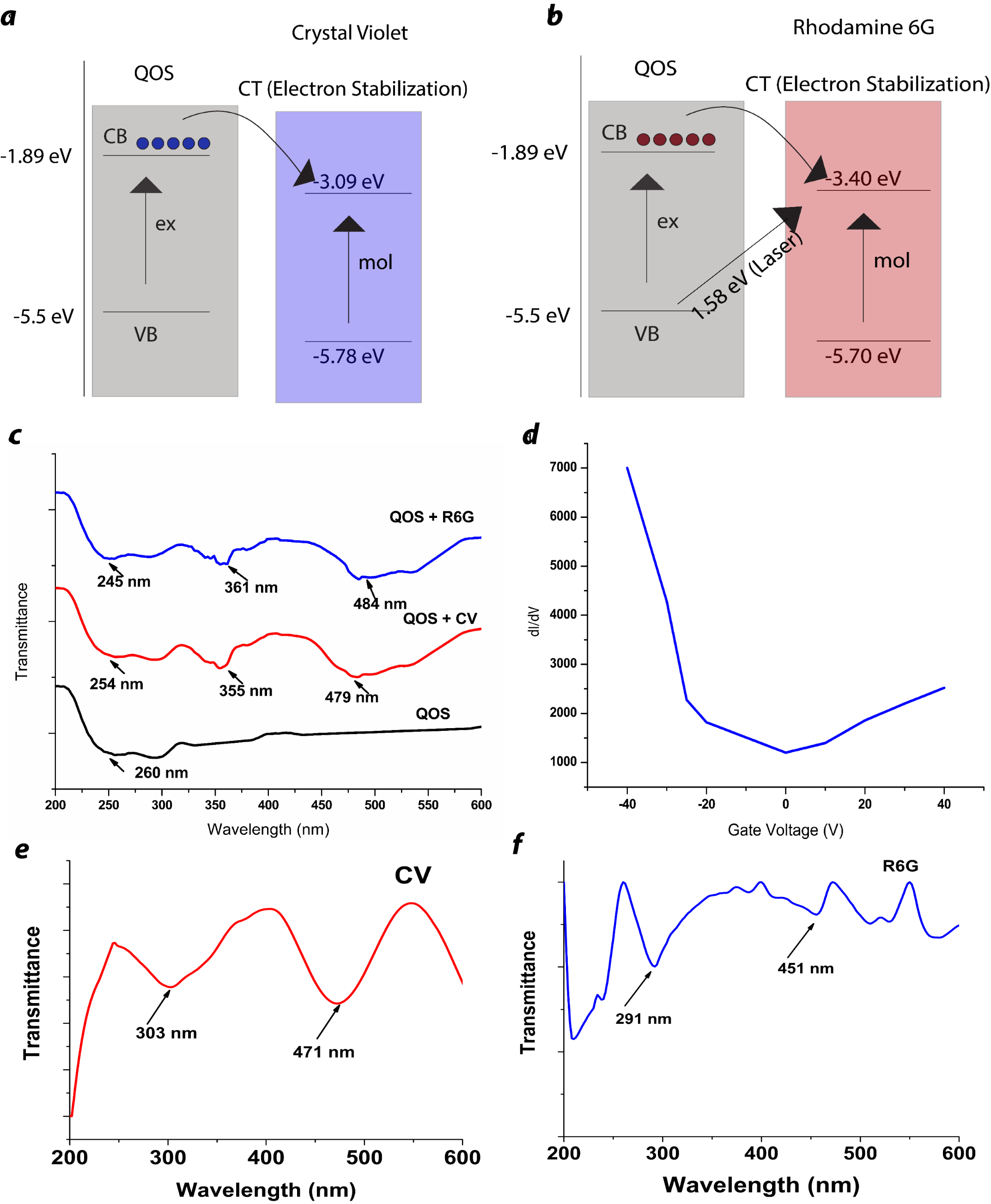


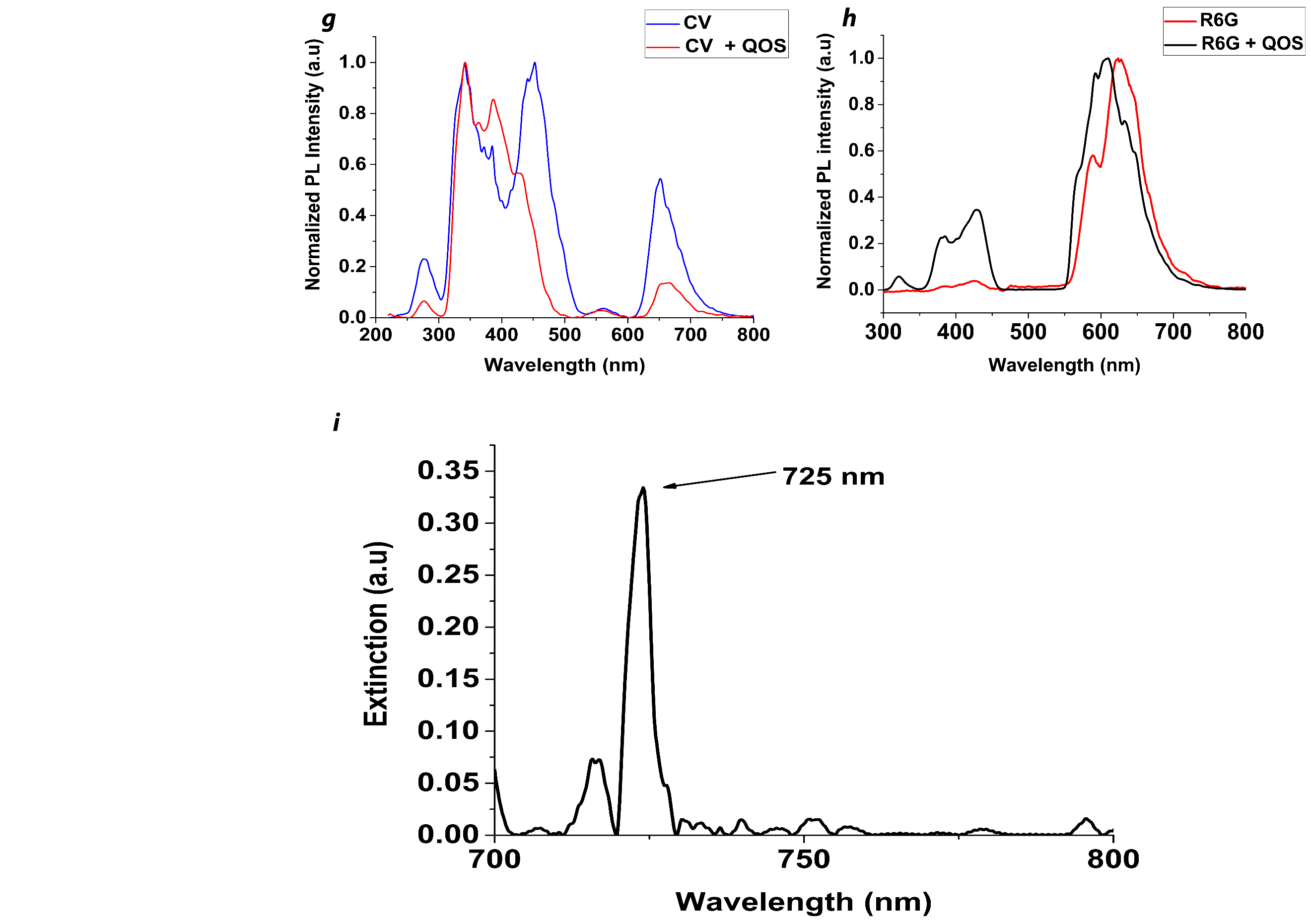


**Supplementary Figure 6: Proposed mechanism of SERS enhancement of analyte molecules adsorbed on QOS a) Charge transfer processes involved in enhancement of CV molecule b) Charge transfer processes involved in enhancement of R6G molecule c) Investigation of interaction between analyte molecules and QOS using UV-Visible transmission measurements d) Differential conductance measurements to prove the presence of inelastic phonon scattering leading to enormous SERS enhancement e)UV-visible transmission measurements of CV f) UV-visible transmission measurements of R6G. Photoluminescence spectra to confirm charge transfer transitions between analyte and QOS g) CV and QOS h) R6G and QOS i) LSPR spectra of QOS to confirm the presence of surface plasmon resonant to the Raman excitation wavelength (785 nm)**

**Supplementary Note 5:** The plasmon resonance of semiconductors is highly dependent on the charge density. Generally, the plasmon resonance of organic semiconductors lies in infrared region, hence the excitation wavelength of 785nm has a great influence on the enhancement efficiency of QOS. The LSPR spectra of QOS is shown in figure S6 i. It can be inferred from the figure S6i that the LSPR of QOS is present at 725 nm, thus validating the utilization of 785nm as the Raman excitation wavelength. Further, the presence of nitrogen atoms in the carbon lattice helps in improving the plasmon propagation length, thereby leading to a high SERS enhancement^9–11^.

In addition, due to the 3D structure of the QOS, another factor that could contribute to the increased enhancement is near field scattering efficiency based on Mie theory^12^. Apart from plasmon resonance, the enhancement mechanism unique to organic semiconductor is exciton resonance^13^. Excitons are electron-hole pairs created because of optical excitation. The generation of exciton in a semiconductor molecule system tends to deviate because of quantum confinement effects.

Another possible contributor for SERS enhancement in the organic semiconductor system is charge transfer resonance^14^. Charge transfer is generally associated with phonon-induced charge transport between the semiconductor and the analyte molecule, which changes the polarizability of the molecule, subsequently increases the Raman signal^15^. The relationship between molecular adsorption and charge transfer between analyte and QOS, was studied using photoluminescence spectra presented in figure S6 g, h for CV and R6G respectively. On observing the spectra, it should be noted that the PL intensity significantly drops on interaction with QOS. This trend is observed in both CV and R6G, although the decrease in intensity for R6G adsorption is lower. This minor intensity drop suggests that the presence of resonant charge transfer in R6G and a non-resonant charge transfer in CV ^16^. Further, comparing the PL intensity of the molecule and molecule/ QOS complex, it can be observed that the molecule/QOS complex exhibit decreased PL intensity. The decreased PL intensity implies the presence of exciton quenching happening as a result of electron transfer between molecule and QOS^17^. The other evidence of charge transfer observed from the PL spectra in figure S6 g, h is the blue shift of CV peak by 25 meV and R6G by 65 meV. In addition, we also observe a narrowing of the peak in both CV (FWHM = 51) CV + QOS (FWHM = 47) and for R6G (FWHM = 85) R6G +QOS (FWHM = 54). The blue shift of PL along with the narrowing of peaks, may be attributed to alterations in exciton recombination leading to efficient charge transfer ^18,19^. On establishing the presence of charge transfer between analyte and QOS, further explanation on the possible charge transfer mechanism is discussed below.

The possible pathways of charge transfer in the molecule-QOS system for CV are shown in figure S6 a and R6G shown in figure S6 b. In the CV-QOS system, the charge transfer is mainly by electron stabilization. The electron in QOS is transferred from ground state to excited state upon incident laser light. Then, the excited electron is transferred to the adsorbed CV molecule through electron stabilization by forming organic–molecule interfacial states. Consequently, the electron is transported to ground state, emitting a Raman photon. In the case of R6G –QOS system, apart from charge transfer through electron stabilization, the laser light used (1.58eV) also contributes to electron transfer between the organic-molecule interface since the 785 nm is the resonant wavelength. Additionally, the use of a resonant wavelength accelerates the charge transfer mechanism. Another mechanism contributing to SERS enhancement is charge transfer through vibronic coupling. The SERS spectra in Figure 4 a b shows an intense peak corresponding to 612 cm^-1^ and Figure 4 e f shows a peak at 778 cm^-1^. These peaks correspond to in-plane and out-of-plane bending motion, consistent with charge transfer through vibrionic coupling^20^. The presence of peaks associated with vibrionic coupling strongly suggests the predominance of ground state charge transfer, consistent with observations in other carbon-based SERS systems^21–23^. The decrease in the intensity of these peaks with decreasing concentration indicate that charge transfer is strongly dependent on molecular adsorption and orientation of the molecule. The relative low intensity at femtomolar concentration indicates that the aromatic rings in CV and R6G were not parallel to QOS surface ^20^.

Another factor influencing the enhancement efficiency of QOS is the particle size. It can be inferred from figure 4 c, d, g, h that the small QOS possess a higher enhancement efficiency compared to Large QOS. This observation is consistent with previously reported literature on semiconductor materials such as ZnO which exhibits size dependent charge transfer enhancement^24^. Further, when the particle size reaches the order of Bohr exciton radius, the deviation between the excited energy level of the molecule-QOS system and the ground state energy level increases with particle size. Hence, small QOS with a median particle size of 3.5 nm possess a higher enhancement efficiency due to smaller energy difference between the excited and the ground state energy levels.

UV-Visible transmission measurements for CV, R6G molecules interacting with QOS in a solution were performed to study the interaction of probe molecules with QOS. Earlier studies employed similar method to get a better understanding of SERS enhancement by carbon-based organic semiconductor probes^25^. In the transmission spectra in Figure S6c, with the addition of analyte molecules there is increase in absorption, indicated by the peaks at 355 nm and 479 nm for CV and 361 nm and 484 nm for R6G. The UV-visible transmission measurements of CV presented in figure S6e shows distinct peaks at 303 nm and 471 nm and for R6G the peaks appear at 291 nm and 451 nm consistent with reported literature^25^. These peaks are considerably red-shifted on interaction with QOS. The increase in absorption in combination with the red-shifting of the absorption peaks implies the presence of probability of electron transition between analyte molecules and QOS. From the transmission spectra, we can observe a blue shift in the absorption peak of QOS when molecular interaction happens. Besides, the molecular adsorption to QOS, leads to a significant shift in the HOMO-LUMO gaps which is confirmed by a blue shift in transmission of QOS after interaction with analyte molecules. The analyte molecules being probed are cations and connects with electron density of QOS by electrostatic interaction through production of image charges^26,27^. Further, the production of image charge shifts the molecular levels thus decreasing the HOMO-LUMO gaps.

The presence of a V-shaped differential conductance curve as shown in figure S6d with a dip near the fermi level suggests the presence of phonon assisted inelastic tunneling consistent with previously reported literature ^28^. In addition, the presence of V-shaped differential conductance indicates a change in local density of states, leading to a larger charge carrier asymmetry. Further, the presence of nitrogen atoms in the carbon lattice, enhances the electron localization around the nitrogen atoms. The enhanced electron localization along with the shift in Dirac point due charge carrier asymmetry and the presence of phonon assisted inelastic tunneling is essential to generate additional charge carriers, which further transforms the charge transfer efficiency of organic semiconductors. Studies have shown that presence of heteroatoms such as nitrogen in the graphene lattice shifts the fermi energy level close to LUMO band of analyte which results in exceptional Raman enhancement.

**Supplementary Table 2: Global DNA hypermethylation levels obtained by SERS and standard colorimetric assay**

|  | QOS based SERS sensor | Colorimetric assay |
| --- | --- | --- |
| Fibroblast | 2.730281066 | 2.64251 |
| Breast Cancer | 4.431938239 | 5.45311 |
| Pancreatic Cancer | 7.464958845 | 7.82156 |
| Lung Cancer | 25.58581672 | 26.38124 |

References

1. Tang, Y., Su, Y., Yang, N., Zhang, L. & Lv, Y. Carbon nitride quantum dots: A novel chemiluminescence system for selective detection of free chlorine in water. *Anal. Chem.* **86**, 4528–4535 (2014).

2. Li, Y. *et al.* Nitrogen-doped graphene quantum dots with oxygen-rich functional groups. *J. Am. Chem. Soc.* **134**, 15–18 (2012).

3. Yadav, R. & Dixit, C. K. Synthesis, characterization and prospective applications of nitrogen-doped graphene: A short review. *J. Sci. Adv. Mater. Devices* **2**, 141–149 (2017).

4. Zafar, Z. *et al.* Evolution of Raman spectra in nitrogen doped graphene. *Carbon N. Y.* **61**, 57–62 (2013).

5. He, S., Turnbull, M. J., Nie, Y., Sun, X. & Ding, Z. Band structures of blue luminescent nitrogen-doped graphene quantum dots by synchrotron-based XPS. *Surf. Sci.* **676**, 51–55 (2018).

6. Zhu, C. *et al.* Negative induction effect of graphite N on graphene quantum dots: Tunable band gap photoluminescence. *J. Mater. Chem. C* **3**, 8810–8816 (2015).

7. Kroupa, D. M. *et al.* Tuning colloidal quantum dot band edge positions through solution-phase surface chemistry modification. *Nat. Commun.* **8**, 2–9 (2017).

8. Lombardi, J. R. & Birke, R. L. Theory of surface-enhanced raman scattering in semiconductors. *J. Phys. Chem. C* **118**, 11120–11130 (2014).

9. Jablan, M., Buljan, H. & Soljačić, M. Plasmonics in graphene at infrared frequencies. *Phys. Rev. B - Condens. Matter Mater. Phys.* **80**, 1–7 (2009).

10. Koppens, F. H. L., Chang, D. E. & García De Abajo, F. J. Graphene plasmonics: A platform for strong light-matter interactions. *Nano Lett.* (2011). doi:10.1021/nl201771h

11. Novko, D. Dopant-induced plasmon decay in graphene. 1–17

12. Lombardi, J. R. The theory of surface-enhanced Raman scattering on semiconductor nanoparticles; Toward the optimization of SERS sensors. *Faraday Discuss.* **205**, 105–120 (2017).

13. Voznyy, O., Sutherland, B. R., Ip, A. H., Zhitomirsky, D. & Sargent, E. H. Engineering charge transport by heterostructuring solution-processed semiconductors. *Nat. Publ. Gr.* **2**, (2017).

14. Zheng, Z. *et al.* Semiconductor SERS enhancement enabled by oxygen incorporation. *Nat. Commun.* (2017). doi:10.1038/s41467-017-02166-z

15. Alessandri, I. Enhancing raman scattering without plasmons: Unprecedented sensitivity achieved by TiO2shell-based resonators. *J. Am. Chem. Soc.* **135**, 5541–5544 (2013).

16. Katayama, K., Shibamoto, K. & Sawada, T. Direct observation of ultrafast charge transfer in relation to the surface enhanced Raman scattering activation detected by transient reflecting grating spectroscopy. *Chem. Phys. Lett.* **345**, 265–271 (2001).

17. Garcia-Basabe, Y. *et al.* Ultrafast charge transfer dynamics pathways in two-dimensional MoS2-graphene heterostructures: A core-hole clock approach. *Phys. Chem. Chem. Phys.* **19**, 29954–29962 (2017).

18. Buscema, M., Steele, G. A., van der Zant, H. S. J. & Castellanos-Gomez, A. The effect of the substrate on the Raman and photoluminescence emission of single-layer MoS2. *Nano Res.* **7**, 1–11 (2014).

19. Ago, H. *et al.* Controlled van der Waals epitaxy of monolayer MoS2 triangular domains on graphene. *ACS Appl. Mater. Interfaces* **7**, 5265–5273 (2015).

20. Cong, S. *et al.* Noble metal-comparable SERS enhancement from semiconducting metal oxides by making oxygen vacancies. *Nat. Commun.* **6**, 1–7 (2015).

21. Wang, L., Zhang, Y., Yang, Y. & Zhang, J. Strong Dependence of Surface Enhanced Raman Scattering on Structure of Graphene Oxide Film. *Materials (Basel).* **11**, 1199 (2018).

22. Balasubramanian, K., Zuccaro, L. & Kern, K. Tunable enhancement of Raman scattering in graphene-nanoparticle hybrids. *Adv. Funct. Mater.* **24**, 6348–6358 (2014).

23. Ling, X., Moura, L. G., Pimenta, M. A. & Zhang, J. Charge-transfer mechanism in graphene-enhanced Raman scattering. *J. Phys. Chem. C* **116**, 25112–25118 (2012).

24. Sun, Z., Zhao, B. & Lombardi, J. R. ZnO nanoparticle size-dependent excitation of surface Raman signal from adsorbed molecules: Observation of a charge-transfer resonance. *Appl. Phys. Lett.* **91**, 1–4 (2007).

25. Feng, S. *et al.* Ultrasensitive molecular sensor using N-doped graphene through enhanced Raman scattering. *Sci. Adv.* **2**, 1–12 (2016).

26. Pham, V. D. *et al.* Molecular adsorbates as probes of the local properties of doped graphene. *Sci. Rep.* **6**, 1–8 (2016).

27. Pham, V. D. *et al.* Electronic interaction between nitrogen-doped graphene and porphyrin molecules. *ACS Nano* **8**, 9403–9409 (2014).

28. Lv, R. *et al.* Nitrogen-doped graphene: Beyond single substitution and enhanced molecular sensing. *Sci. Rep.* **2**, 1–8 (2012).
